# Image of the month: Measles inclusion body encephalitis

**DOI:** 10.5414/NP301283

**Published:** 2020-06-08

**Authors:** Iban Aldecoa, Iván Archilla, Laura Herrero, Felipe Garcia, Berta Torres, Carles Gaig, Sara Fernández, María Ángeles Marcos, Ellen Gelpi

**Affiliations:** 1 Department of Pathology, Biomedical Diagnostic Centre (CDB), Hospital Clinic, University of Barcelona,; 2 Neurological Tissue Bank of the Biobank-Hospital Clinic, IDIBAPS,; 3 Infectious Diseases Service, HIV Unit, Hospital Clinic de Barcelona,; 4 Department of Neurology, Neuroscience Institute, Hospital Clinic de Barcelona,; 5 Medical Intensive Care Unit, Hospital Clinic, University of Barcelona,; 6 Department of Microbiology, Biomedical Diagnostic Center (CDB), Hospital Clinic,; 7 Institute for Global Health (ISGlobal), Barcelona, Spain, and; 8 Division of Neuropathology and Neurochemistry, Department of Neurology, Medical University of Vienna, Austria

**Keywords:** measles encephalitis, nuclear inclusion body, immunosuppression, HIV

## Abstract

No abstract available.

We present the postmortem neuropathological findings of a 37-year-old HIV-infected man presenting with stupor and super-refractory status epilepticus. HIV infection had been diagnosed 3 years before in the context of unexplained weight loss and prolonged fever. Viral load was 555,000 copies/mL, and his CD4 count was 9 cells/mm^3^. He immediately started antiretroviral treatment with good virological but poor immunological response. Three months later, he developed malaise, low-grade fever, and diarrhea. *Mycobacterium avium intracellulare* (MAI) was isolated in feces and sputum. Despite virologic control of HIV infection, CD4 count remained below 100 cells/mm^3^, and 22 months after MAI treatment he was admitted to the hospital with increased bowel movements, severe hypocalcemia, hypomagnesemia, and hypokalemia. He had also a severe corticosteroid-induced osteoporosis and mild hypothyroidism. After appropriate treatment, he improved progressively. However, 6 months after discharge he consulted the HIV out-patient clinic for ~ 5 days of general malaise, restlessness, tremor, and insomnia. Physical examination revealed distal tremor and akathisia, which was initially attributed to a recent increase of levothyroxine. His state worsened during the following days, and he developed mental cloudiness, dysarthria and dysphagia, bilateral reactive mydriasis, and 38 °C fever. He had tachycardia and tachypnea, left pulmonary hypophonesis, and dry crackles. He rapidly developed acute respiratory failure due to aspiration that required intubation and mechanical ventilation. At that moment, the patient presented facial myoclonus and right hemiparesis with pyramidal signs. A brain CT was normal, and lumbar puncture showed an acellular cerebrospinal fluid (CSF). Initial brain MRI revealed T2 signal and FLAIR hyperintensities predominantly in the midbrain tegmentum without gadolinium enhancement, suggestive of Wernicke encephalopathy. High-dose parenteral thiamine was administered without improvement, and the patient became stuporous with persistent facial myoclonus. He developed a super-refractory status epilepticus for 4 weeks with EEG showing diffuse delta-theta slowing with continuous and recurrent bilateral frontotemporal epileptic seizures despite treatment with multiple anti-epileptic drugs, and thiopental- and ketamine-induced coma. All microbiological tests from CSF were negative, including *Cryptococcus neoformans*, HSV1 and 2, EBV, CMV, HHV6, enterovirus, JC virus, *Toxoplasma gondii*, and *Tropheryma whipplei*. Mycobacterium PCR and 16S rRNA gene by PCR were also negative. A bronchoalveolar lavage did not result in any positive microbiological test, including *Pneumocystis jirovecii*, respiratory virus, *S. pneumoniae*, *L. pneumophila*, atypical bacteria, and fungi. Blood cultures were also negative. 

Subsequent brain MRI showed progression of T2 signal and FLAIR hyperintensities without contrast enhancement into the brainstem and cerebellum with spreading to both brain hemispheres with multifocal cortical and subcortical grey matter lesions including basal ganglia and thalamus (Figure 1). These hyperintense lesions presented abnormal restricted diffusion on ADC maps and were compatible with cytotoxic edema and suggested an underlying encephalitic process. Onconeuronal and surface autoantibodies in CSF were also negative. In the absence of an infectious cause, high-dose steroids and immunoglobulins were administered without clinical improvement. A brain biopsy was suggested but was not possible to perform. Despite all treatments, the neurological status of the patient in refractory status epilepticus worsened progressively, and electrolyte imbalance led to death. 

Neuropathological examination revealed a diffuse polioencephalitis/encephalopathy with diffuse and prominent cortical and subcortical gliosis and focal microglial nodules. Some multinucleated cells were detected in cortical areas, the brainstem, and in the cerebellar cortex. The most striking feature was the presence of abundant large intranuclear inclusions that were already visible on HE-stained sections as enlarged, bright eosinophilic glial and neuronal nuclei, highly suggestive of viral inclusions. Multinucleated cells also harbored intranuclear eosinophilic inclusions. These were prominent in the limbic system, in brainstem regions and also in the cerebellum, here mostly involving Bergmann glia, and less frequently, granular neurons (Figure 2A, B). Depletion of Purkinje cells was also a prominent feature. There were only discrete inflammatory infiltrates with moderate parenchymal CD3+ T cells in affected areas and mild perivascular lymphocytic cuffing. Immunohistochemistry revealed abundant measles viral antigens in affected cells (Figure 2C). PCR for measles virus RNA from fresh brain sample was also positive, confirming the diagnosis of measles inclusion body encephalitis (MIBE). In addition, multiple subacute hypoxic-ischemic lesions were observed in cortical areas. Moreover, lesions in mammillary bodies, paraventricular region, and midbrain colliculi with abundant macrophages and capillary proliferation were suggestive of additional Wernicke encephalopathy. 

In sum, we present a relatively fulminant case of MIBE. This is a rare subacute viral encephalitis that progresses over weeks to months and is caused by a single-stranded RNA virus of the paramyxovirus family [1]. The primary infection is usually acquired by inhalation, and presents with a characteristic cutaneous rash and fever [1]. Encephalitis may occur either during the primary infection or as an acute postinfectious immune-mediated complication, or may develop within months or even years after the initial infection in form of either inclusion body encephalitis or subacute sclerosing panencephalitis (SSPE) [1, 2]. MIBE affects usually immunodepressed patients with impaired cellular immunity, such as HIV [3] or rarely after stem-cell transplantation [4]. It presents with a variety of neurological symptoms and has in most cases a fatal evolution within weeks, despite anti-viral (ribavirin) and supportive treatment [1, 2]. While MIBE is often accompanied by a poor immunological response and nonrestricted virus replication, SSPE has a more chronic presentation, in months and years, may encompass a progressive dissemination of defective virus replication (e.g., due to mutations in different viral proteins), and may be associated with a florid humoral and cellular immune response [2]. In addition, mutations in the measles envelope glycoproteins, such as the fusion protein, may increase the CNS tropism of the virus and promote neuronal spread [5, 6]. 

The number of cases of measles encephalitis has increased over the past 10 – 15 years in developed countries due to an increase in measles infection, most likely due to insufficient vaccination [1]. Paradoxically, our patient was correctly vaccinated and had a positive IgG measles serology 3 years before he developed the neurological complication. However, dysfunction in cellular immunity, due to severe immunosuppression, and the possibility of waning antibodies could have made him susceptible to measles infection. The possibility of a brain biopsy should be considered in patients with unclear subacute neurological disease, particularly in immunosuppressed and unvaccinated subjects coming from endemic/epidemic countries. Characteristic neuropathological features as presented here, combined with polymerase chain reaction and immunohistochemical studies of brain tissue may allow an early diagnosis of subacute measles encephalitis and provide a basis for early and targeted anti-viral therapy to improve clinical outcome. 

## Funding 

There was no specific funding for this work. 

## Conflict of interest 

The authors report no conflict of interest. 

**Figure 1.**
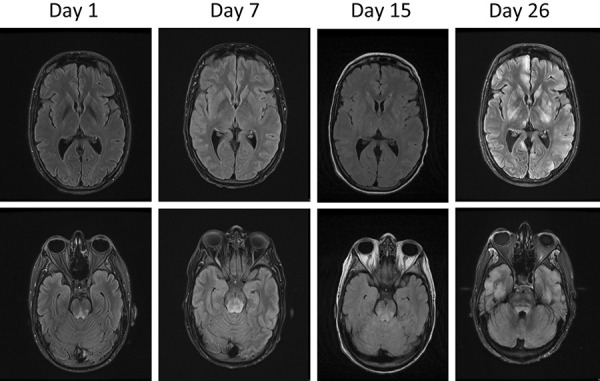
Brain MRI axial FLAIR (fluid-attenuated inversion recovery) sequence findings at day 1, 7, 15, and 26 after ICU admission showing severe progression of signal alterations in the brainstem, cortical areas, and basal ganglia.

**Figure 2.**
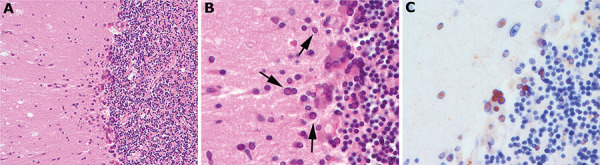
Histological findings. A, B: Cerebellar cortex with diffuse gliosis in the molecular layer and frequent large eosinophilic intranuclear inclusions that are already visible on HE-stained sections (arrows). There are also multinucleated cells that harbor abundant eosinophilic inclusions. There are no prominent inflammatory infiltrates, but some microglial nodules. Immunohistochemistry reveals abundant measles virus antigens (C, brown signal). According to the literature, neurons, astrocytes, and oligodendrocytes are usually infected by the virus. Multinucleated cells have been suggested to be a result of a fusion of virus-containing glial cells [7, 8]. Magnifications: A: × 200; B, C: × 600.
